# Durum wheat and allelopathy: toward wheat breeding for natural weed management

**DOI:** 10.3389/fpls.2013.00375

**Published:** 2013-09-24

**Authors:** Mariagiovanna Fragasso, Anna Iannucci, Roberto Papa

**Affiliations:** Consiglio per la Ricerca e la Sperimentazione in Agricoltura, Cereal Research CentreFoggia, Italy

**Keywords:** allelopathy, wheat, durum wheat, allelochemical, crop allelopathy, weed management, sustainability

## Abstract

Wheat-derived foodstuffs represent about one-fifth of the calories consumed by humans worldwide. Bread wheat (*Triticum aestivum* L.) is one of the most important crops throughout the world, and it has been extensively studied for its allelopathic potential. In contrast, for allelopathy in durum wheat (*Triticum turgidum* ssp. *durum*), our knowledge is partial and fragmentary. Through highlighting recent advances in using allelopathy as a crop-breeding tool, we provide an overview of allelopathy in *Triticum *spp., to stimulate further coordinated breeding-oriented studies, to favor allelopathy exploitation for the sustainable cultivation of wheat, and in particular, to achieve improved biological weed control.

## INTRODUCTION

Competition among plants has been divided into two different biological phenomena: competition in terms of the “removal” of shared or limited resources, such as space, light, water, and/or nutrients; and allelopathy, in terms of chemical interactions between plants (Olofsdotter et al., 2002). Allelopathy appears to have been first noted by the Greek philosopher and botanist Theophrastus as early as 2300 BP (Weir et al., 2004), and it is defined as “the inhibitory effect of a plant (donor) on other plants (receivers) through the release of toxic chemicals produced by the donor plant into the environment that affect a receiving susceptible species” (Olofsdotter et al., 2002). These competitive abilities are genetically controlled and can be affected by the environment in which a plant grows (Olofsdotter et al., 2002). Chemical compounds that have allelopathic effects can have adaptive roles, such as for plant defense and nutrition, and soil fertility (as in the regulation of the soil biota, which affects the organic matter decomposition; Inderjit et al., 2011). Scientific attentiveness to allelopathy as a crop-breeding tool is a consequence of the economic relevance associated with: (i) the development of herbicide-resistant populations in key weed species (Worthington and Reberg-Horton, 2013); (ii) the need for improved weed suppression in organically grown cereals (Hoad et al., 2012); and (iii) the need for low cost weed control for smallholder farmers, especially in developing countries (Toure et al., 2011). For these reasons, an advance in allelopathic potential in crop varieties might have a remarkable impact on both low-input and high-input agricultural management systems (Kim and Shin, 2003).

With 620 million tons of wheat grain produced annually worldwide, wheat represents about one-fifth of the calories consumed by humans (United Nations, 2006). Roughly 95% of this wheat is obtained from the hexaploid species *Triticum aestivum *L. ssp.* aestivum* (bread wheat), which is used for making bread, cookies and pastries; the remaining 5% is obtained from the tetraploid durum wheat (*Triticum turgidum *ssp*. durum *Desf. em. Husn.). Bread wheat is also the main ingredient for pizza, and several cake doughs. The European Union, China, India, The United States of America, and the Russian Federation are the main wheat producers (FAO, 2011). Durum wheat is used for the production of pasta, semolina, and couscous, and in some areas of the world, also for various types of bread. The major durum wheat production areas are in the European Union (e.g., it is the main cereal crop in Italy), Canada, Syria, The United States of America, Algeria, and Morocco, and the minor production areas include the Russian Federation, Turkey, Tunisia, Mexico, and India (De Vita et al., 2007a,b).

In this mini review, we explore the present knowledge of wheat allelopathy, to illustrate its potential for the control of weeds. We underline the partial and fragmentary evidence, particularly in the case of durum wheat. The aim is thus to stimulate further coordinated breeding-oriented studies and to favor allelopathy exploitation for the sustainable cultivation of wheat.

## CEREAL ALLELOPATHY IN A BREEDING-ORIENTED PERSPECTIVE

A breeding-oriented framework of allelopathy studies requires the characterization of the genetic basis of allelopathy and its diversity, along with the definition of the breeding strategies. In 1996, the International Allelopathy Society includes in allelopathic phenomena any process involving secondary metabolites produced by plants, microorganisms, algae, and fungi that influence the growth and development of agricultural and biological systems. Allelopathy is associated with the release of chemical compounds from plants that “suppress the growth and establishment of other plants in their vicinity” (Inderjit et al., 2011), determining the patterning densities and distribution of various species. In addition, these chemicals can help plants to reinforce their protection system against biotic and abiotic stress, and to promote the regulation of nutrient transformation and absorption, and soil fertility (Inderjit et al., 2011; Jabran and Farooq, 2013). Thus, allelopathy might have a significant role in the determination of the diversity of plant communities (Inderjit et al., 2011). To exemplify the adaptive role of allelopathy, Putnam and Duke (1974) observed in cucumber that the ancestors of the present crops possessed high allelopathic activity. However, standard agronomic practices (e.g., herbicide application) have reduce the need for competition with weeds and the associated selection pressure in plant breeding programs, which could thus explain the competitive loss in the modern cultivar (Bertholdsson, 2004; Wolfe et al., 2008). In contrast with this trend, in an analysis of Swedish wheat cultivars, Bertholdsson (2007) underlined how landraces and old cultivars were less allelopathic than modern varieties.

From a breeding perspective (Bertholdsson, 2005; Weih et al., 2008; Prohens, 2011), the application of genomic approaches is crucial for the identification and characterization of genes with ecological and evolutionary relevance (Ungerer et al., 2008). Effectively, cereal crops including rice, sorghum, wheat, rye, maize, and barley, show strong allelopathic activities, with the potential for identification of the molecular dissection of this trait to promote allelopathy-oriented crop improvements (Jabran and Farooq, 2013). For many major crop species (e.g., rice, wheat, oat, barley, rye), a high level of genotypic differences is seen within species (Fay and Duke, 1977; Burgos et al., 1999; Ahn and Chung, 2000; Wu et al., 2000b, 2001b; Bertholdsson, 2004; Jung et al., 2004; Seal et al., 2004; Ahn et al., 2005; Grimmer and Masiunas, 2005; Ma, 2005; Reberg-Horton et al., 2005; Zuo et al., 2007; Saffari and Torabi-Sirchi, 2011).

Enhancement of the competitive ability of a crop for weed suppression through allelopathic potential using plant breeding requires efficient phenotyping strategies, both at field evaluation and under controlled conditions. Indeed, to undertake a breeding program, a good screening technique needs to be developed, the genetic variability for the target trait should be accessible in the germplasm, and the genetic control of the desired phenotype should be defined.

In the following sections, we briefly review the case for wheat: the weed competitive arena, the allelopathy phenomena, and some genetic aspects.

## THE WHEAT/WEED ENVIRONMENT

Weeds are responsible for heavy yield losses in wheat because of their competition for water, nutrients, and light, and also, as shown for *Avena fatua* L. (wild oats), for their toxic effects. The distribution of grass weeds in cereal crops is usually patchy. Such weed patches can be quite steady over a number of growing cycles, as can weeds such as wild oat, that can cause a 2% yield loss in cereal crops even at low density (one or two plants per m^2^; Carrara et al., 2004). Herbicide treatments achieve the best results in terms of reducing the weed biomass, followed by hoeing and harrowing (Garcìa-Martìn et al., 2007). Even if durum wheat responds differently to some herbicides compared to bread wheat, using conventional farming systems, both crop species require the application of considerable quantities of herbicides, with the same amounts spread throughout the field (Soltani et al., 2011). These are necessary to control the major monocotyledonous and dicotyledonous weeds, such as *Avena fatua* L. (wild oat), *Lolium perenne* L. (annual ryegrass), *Phalaris* spp. L. (canary grass), *Alopecurus myosuroides* Huds. (blackgrass), and *Galium aparine* L. (cleaverwort; Barberi et al., 1997; Carrara et al., 2004; Bertholdsson et al., 2012), as reported by Garcìa-Martìn et al. (2007) and Barberi et al. (1997).

The other most widespread weeds in wheat crops are reported in **Table 1**. This list is of particular interest as a reference if we consider that in an experimental design of allelopathic studies, it is important to conveniently select a panel of weeds that are locally relevant (as receiver species), as a function of the induced losses in wheat production, and thus of the economic significance (Worthington and Reberg-Horton, 2013).

**Table 1:** A list of widespread weeds in wheat crops, with their scientific names, common names, and global geographical distributions. The global distribution is reported in accordance with the AgroAtlas (Afonin et al., 2008; ; n.r., not reported in the AgroAtlas).

Scientific name	Common name	Global geographical distribution
*Achillea millefolium* L.	Bloodwort	Europe; Asia, Japan, China; Northern America; Australia, and New Zealand
*Amaranthus retroflexus* L.	Careless weed	North and South America; middle and southern Europe; Mediterranean area; Asia Minor; Iran; China; Japan; Mongolia; northern Africa
*Anagallis arvensis* L.	Scarlet pimpernel	n.r.
*Anthemis arvensis* L.	Corn chamomile	European part of Russia, Caucasus; Siberia, Central Asia; Scandinavia, Mediterranean
*Avena sterilis *L.	Animated oat	n.r.
*Capsella bursa-pastoris* L.	Shepherd’s purse	Cosmopolitan (this weed is found in all parts of the world except tropical regions)
*Cardamine hirsuta* L.	Hairy bittercress	n.r.
*Chenopodium album* L.	Common lambsquarters	Cosmopolitan (this weed is found in all parts of the world)
*Cichorium intybus* L.	Chicory	n.r.
*Cirsium arvense* (L.) Scop	Creeping thistle	Europe; Western Asia; North America
*Convolvulus arvensis* L.	Field bindweed	Almost cosmopolitan (Far East, Central Asia, Western Europe, Asia, Northern Africa, Northern and South America)
*Equisetum arvense* L.	Field horsetail	Europe, Caucasus, Siberia, the Himalaya, Central China, Japan; North America; New Zealand
*Lactuca serriola* L.	Prickly lettuce	Western Europe (southward of 55 degrees latitude); western Asia; northern Africa
*Malva sylvestris* L.	Common mallow	n.r.
*Polygonum aviculare* L.	Prostrate knotweed	Cosmopolitan (this weed is found in all parts of the world)
*Polygonum persicaria* L.	Smartweed	n.r.
*Rumex crispus* L.	Curly dock	Europe; North Africa; Turkey; northern Iran, eastern-central Asia; North America
*Rumex obtusifolius* L.	Bitter dock	n.r.
*Silene inflata* Sm.	Catchfly	n.r.
*Sinapis arvensis* ssp. *arvensis*	Wild mustard	Europe; North Africa; Asia Minor, Iran, Afghanistan; North America
*Silybum marianum* (L.) Gaertn.	Milk thistle	n.r.
*Solanum nigrum* L.	Black nightshade	European part of the Former Soviet Union, the Caucasus,. Distributed also in Scandinavia, Europe, Mediterranean; Central Asia, Siberia, Far East, Iran, India, China, Japan; North Africa; North America
*Sonchus asper *L. (Hill)	Prickly sowthistle	Almost all Western Europe, Asia Minor, Iran, Afghanistan, the Himalaya, Mongolia, South and East Asia, North and South America, Australia
*Stellaria media* L.	Common chickweed	Europe; Asia; North America
*Vaccaria hispanica* (Mill) Rauschert	Cowherb	n.r.
*Veronica hederifolia* L.	Ivy-leaved speedwell	n.r.
*Veronica persica* Poir.	Winter speedwell	n.r.
*Vicia villosa* Roth	Winter vetch	Europe; Asia; North Africa; North America

## ALLELOPATHY AND WHEAT

**Table 2** summarizes the various investigations into wheat allelopathy, to the best of our knowledge. Studies on bread wheat allelopathy have included: (i) allelopathy against other crops, weeds (Wu et al., 1999, 2000a,b, 2001a); (ii) isolation and identification of allelopathic agents (Wu et al., 2001a,b,c); (iii) wheat autotoxicity (Wu et al., 2001a, 2007a); (iv) management of residues (Wu et al., 2001a); and (v) genetic variations and genetic markers (Wu, 2005; Wu et al., 2008).

**Table 2:** Overview on the major studies carried out relating to allelochemicals, allelopathic genes, field evaluations, allelopathic bioassays, and breeding programs in wheat.

Aspect investigated	Bread wheat	Durum wheat
Allelochemicals	Short-chain fatty acids (Tang and Waiss, 1978; Lynch et al., 1980; Hairston et al., 1987)	Iannucci et al. (2012)
	Phenolic acids (Chaves das Neves and Gaspar, 1990; Gaspar and Chaves das Neves, 1995; Wu et al., 1999, 2001b,c,d)	
	Hydroxamic acids (Copaja et al., 1991; Nicol et al., 1992; Wu et al., 1999; Stochmal et al., 2006)
	Chaves das Neves and Gaspar (1990) (naphthoic acid, azelaic acid and 1,2,3,5-tetrabromobenzene)	
	Gaspar and Chaves das Neves (1995) (carboxylic acid methyl esters and triterpenoids)	
Allelopathic genes and genetic markers	Niemeyer and Jerez (1997) (QTLs controlling hydroxamic acid accumulation)	
	Wu et al. (2003) (two major QTLs associated with allelopathy on chromosome 2B) Wu et al. (2008) (reviewed genetic markers associated with wheat allelopathy)	
	Zuo et al. (2012) (QTLs related to allelopathy and weed competitive ability on chromosomes 1A and 2B)	
Field evaluations	Lemerle et al. (1996) (Australia) (*)
	Cousens and Mokhtari, 1998 (Australia) (*)
	Lemerle et al. (2001) (Australia) (*)
	Vandeleur and Gill (2004) (Australia) (*)

	Huel and Hucl (1996) (Canada)
	Mason et al. (2007) (Canada)
	Mason et al. (2008) (Canada)
	Bertholdsson (2005) (Sweden) (**)
	Murphy et al. (2008) (U.S.)
	Acciaresi et al. (2001) (Argentina)
	Blackshaw (1994) (Canada)
	Verschwele and Niemann (1993) (Germany) (°)
	Drews et al. (2009) (Germany) (°)
	Balyan et al. (1991) (India)
	Bertholdsson (2011) (Sweden) (***)
	Champion et al. (1998) (United Kingdom) (•)
	Seavers and Wright (1999) (United Kingdom) (•)
	Korres and Froud-Williams (2002) (United Kingdom) (•)
	Challaiah et al. (1986) (U.S.) (◊)
	Wicks et al. (1986) (U.S.) (◊)
	Wicks et al. (2004) (U.S.) (◊)
Allelopathic bioassays	Wu et al. (2000a) (Australia) (*)	Oueslati (2003) (Tunisia)
	Wu et al. (2000b) (Australia) (*)	Fragasso et al. (2012) (Italy)
	Wu et al. (2003) (Australia) (*)
	Bertholdsson (2005) (Sweden) (**)
	Bertholdsson (2007) (Sweden) (**)
	Niemeyer and Jerez (1997) (Chile)
	Bertholdsson (2011) (Sweden) (***)
	Bertholdsson et al. (2012) (Sweden) (***)
Breeding programs	Coleman et al. (2001) (Australia) (*)
	Mokhtari et al. (2002) (Australia) (*)
	Bertholdsson (2010) (Sweden) (**)

The symbols “*,” “**,” and “***” denote different phases of the same study, from field evaluations to breeding programs, through the allelopathic bioassays.

*The symbols “◊,” “°,” and “•” indicate studies that used the same germplasm.*

Along these lines, the allelopathic activities of wheat varieties change with respect to the major weeds, which indicates the possibility to exploit this characteristic for the selection of allelopathic varieties to be used in integrated weed management. Some of the classes of allelochemicals for wheat allelopathy have also been defined, such as phenolic acids, hydroxamic acids, and short-chain fatty acids. Also, when wheat straw remains on the soil surface, the undesirable impact of the resultant wheat autotoxicity on agricultural production has been noted (for reviews, please see Wu et al., 2001a, 2008).

In contrast, very little is known about durum-wheat allelopathy. In consideration of allelopathy against other crops, Oueslati (2003) studied the two durum-wheat varieties that are commonly used in northern Tunisia, and tested these for toxicity (heterotoxicity) against bread wheat and barley, with an evaluation of the allelopathic potential of various parts of the durum-wheat plants. This study highlighted the role of durum wheat as a donor plant, that may constitute a risk to crop sequences. Additionally, it has been demonstrated that the allelopathy of durum wheat varies with the source of the extracts, whereby the leaf extracts are the most active. This is in agreement with evidence from bread wheat, where aqueous extracts from residues were shown to suppress barley growth (Hozumi et al., 1974). Here, the negative effects of bread wheat residue allelopathy on the growth of other crops were also shown to vary across wheat varieties (Wu et al., 2001a). Indeed, as also shown by Oueslati (2003), the two durum-wheat varieties tested can lead to slight differences in allelopathic effects on barley and bread wheat. Recently, Fragasso et al. (2012) determined the tolerance of seven durum-wheat cultivars to the allelopathic potential of the rhizosphere soil of wild oat. This study was performed in a growth chamber, using durum-wheat varieties that were seeded and grown in control (non-rhizosphere) soil and rhizosphere soil of wild oat. The results showed that the degree of inhibition is more evident for the leaves than for root growth, and that it is cultivar dependent.

Wild oat is a serious weed that can severely affect the survival and productivity of several crops (Jabran et al., 2010). With regard to specific crop–weed allelopathic interactions, the major information relates to an evaluation of the allelopathic potential of wheat on wild oat. Wheat progenitors (different accessions of *Aegilops speltoides* Tausch.) were screened for differential seedling allelopathy on the growth of wild oat and lettuce (Hashem and Adkins, 1998; Quader et al., 2001). The evidence showed that wheat plants release a diversity of allelochemicals into the environment. Hydroxamic acid 2,4-dihydroxy-7-methoxy-1,4-benzoxazin-3-one (DIMBOA) and its decomposition product 6-methoxybenzoxazolin-2-one from bread wheat can inhibit root growth and seed germination of wild oat (Pérez, 1990). The mechanism of action of these molecules appears to be related to the combination of cyclic hemiacetal and cyclic hydroxamic acid, which lead to high bioactivity: “as reactions with the electrophilic ring-opened aldehyde form of the hemiacetal and with a multicentered cation generated from N–O fission are likely to occur with bionucleophiles” (Macías et al., 2006).

More generally, phenolic compounds are one of the main groups of substances involved in bread wheat allelopathy (Wu et al., 2001c). Intriguingly, in studies of wild oat allelopathic potential as a donor plant, phenolic compounds appear to be among the key compounds (Iannucci et al., 2012). Indeed, Fragasso et al. (2012) evaluated the tolerance of wheat to the allelopathic potential of wild oat, and indicated the presence of three potential allelochemicals that belong to this chemical class in the rhizosphere soil of the wild oat: *p*-coumaric acid, syringaldehyde, and vanillin. It is well-known that phenolic compounds can inhibit root elongation and cell division in plants, and can cause changes to cell ultrastructure, thus interfering with the normal growth and development of the whole plant (Li et al., 2010).

## WHEAT ALLELOPATHY: WALKING ON THE GENETIC SIDE

Information on the allelopathic diversity among crop species and varieties can be dated back to ancient times. However, studies on the genetic variability of allelopathy in crop cultivars as a target for breeding are relatively recent (Belz, 2007). Spruell (1984) performed a first evaluation of the varietal allelopathic activity of *Triticum aestivum* L. Hundreds of bread wheat genotypes were shown to vary in their allelopathic activities (Wu et al., 2000b, 2001b,d; Zuo et al., 2007; Saffari and Torabi-Sirchi, 2011). Indeed, Wu et al. (2000b) assessed seedling allelopathy of 453 wheat accessions (from 50 countries) against annual ryegrass, showing different levels of inhibition of root growth of ryegrass, ranging from 9.7 to 90.9%: the continuous distribution of allelopathic activities highlighted among the cultivars indicates a quantitative mode of inheritance. This was confirmed by an assessment of allelopathic compounds produced by the same genotypes. Indeed, in comparison to weakly allelopathic accessions, strongly allelopathic accessions produced significantly higher amounts of allelochemicals (Wu, 2005). Similarly, recently, Fragasso et al. (2012) showed genetic diversity for allelopathic sensitivity among a few varieties of durum wheat.

Direct breeding efforts to improve allelopathy have to date only been reported in rice, where highly allelopathic cultivars are ready to be released (Hu et al., 2008; Kong et al., 2011). Recent technical advances make it possible to locate the genes involved in the control of allelopathic activity and the production of allelochemicals, and to genetically map populations between allelopathic and non-allelopathic accessions, with the final aim being to identify genetic markers associated with allelopathy. In a review on the allelopathic studies in common wheat, Ma (2005) revised the genotypic differences in the allelopathy of wheat toward weeds and other crops. Some effort has been made to map allelopathy genes in wheat (Niemeyer and Jerez, 1997; Wu et al., 2003) and to identified genetic markers (Wu et al., 2008). Niemeyer and Jerez (1997) used wheat aneuploids and wheat substitution lines to suggest the chromosomal location of genes for hydroxamic acid accumulation (4A, 4B, 4D, 5B) and the multigenic control of this character. Wu et al. (2003) used restriction fragment length polymorphism, amplified fragment length polymorphism, and microsatellite markers, and on chromosome 2B, they identified two major quantitative trait loci (QTLs) associated with wheat allelopathy. Wu et al. (2007b) reviewed the genetic markers associated with wheat allelopathy and the plant cytochrome P450s that encode the biosynthesis of wheat allelochemicals. Recently, putative QTLs related to allelopathy and the competitive abilities of weeds were detected on chromosomes 1A and 2B of bread wheat, with possible beneficial insights for the breeding of allelopathic wheat (Zuo et al., 2012). This study also suggested the possibility to identify genetic diversity for these traits also in durum wheat, and the possibility of introgressing the QTLs found in the A and B genomes of bread wheat and durum wheat. Following the common “scale-up” of allelopathic exploitation in crop systems, **Table 2** provides an overview of field and laboratory screening protocols, genetic studies, and breeding efforts that have been undertaken to improve allelopathy and competition in wheat. The discovery of additional fine resolution QTLs that control allelopathy in wheat provides the scientific basis for the development of effective molecular markers to be used in marker-assisted selection for allelopathy (Worthington and Reberg-Horton, 2013). In the most recent breeding program, Bertholdsson (2010) used material that originated from a cross between a Swedish cultivar with low allelopathic activity and a Tunisian cultivar with high allelopathic activity. He analyzed the results of a breeding program with bread wheat that determined the efficiency of selection for allelopathy by assessing the ability of the plants to suppress weeds at the field level: highly allelopathic lines obtained from a cross between allelopathic and non-allelopathic parents suppressed weed biomass by 24% more than the non-allelopathic parent in a dry year (12% more in a wet year; Bertholdsson, 2010).

## IMPROVING WHEAT COMPETITIVE ABILITY, AND THE CASE FOR DURUM WHEAT

Wu et al. (2001a) provided an overview of the beneficial and unfavorable effects of allelopathy in bread wheat. In particular, the positive implications concern direct (allelochemicals produced during the life cycle) and indirect (plant residues) weed suppression. On the other hand, the critical effects were: autotoxicity and the wheat yield decline in crops grown as part of short rotations; and the detrimental effects of residues on the growth of other crops (Bennett et al., 2012). Comparing these important implications and the present knowledge briefly summarized here, it appears clear how far we are from being able to manage the beneficial and unfavorable effects of wheat allelopathy.

If the “cons” of durum-wheat allelopathy are clearly represented by this partial and fragmentary scientific knowledge, then the relatively “clean slate” that we have to cope with at present probably represents a “pro,” given that in the experimental design we have the opportunity to consider also the recent advances in allelopathy studies: the roles of microbes as targets and mediators of allelopathy in plants (Cipollini et al., 2012), the importance of separating allelopathy from resource competition (He et al., 2012; Iannucci et al., 2012); and the “breeding” (Bertholdsson, 2005, 2007; Worthington and Reberg-Horton, 2013) and “ecological genomics” (Ungerer et al., 2008; Weih et al., 2008) dimensions of the allelopathy phenomena.

## Conflict of Interest Statement

The authors declare that the research was conducted in the absence of any commercial or financial relationships that could be construed as a potential conflict of interest.
